# Transcriptional and Post-Transcriptional Mechanisms of the Development of Neocortical Lamination

**DOI:** 10.3389/fnana.2017.00102

**Published:** 2017-11-09

**Authors:** Tatiana Popovitchenko, Mladen-Roko Rasin

**Affiliations:** Neuroscience and Cell Biology, Robert Wood Johnson Medical School, New Brunswick, NJ, United States

**Keywords:** neocortical lamination, mouse neocortex, transcription factors, RNA-binding proteins, post-transcriptional regulation, neurogenesis, pyramidal neuron, alternative splicing

## Abstract

The neocortex is a laminated brain structure that is the seat of higher cognitive capacity and responses, long-term memory, sensory and emotional functions, and voluntary motor behavior. Proper lamination requires that progenitor cells give rise to a neuron, that the immature neuron can migrate away from its mother cell and past other cells, and finally that the immature neuron can take its place and adopt a mature identity characterized by connectivity and gene expression; thus lamination proceeds through three steps: genesis, migration, and maturation. Each neocortical layer contains pyramidal neurons that share specific morphological and molecular characteristics that stem from their prenatal birth date. Transcription factors are dynamic proteins because of the cohort of downstream factors that they regulate. RNA-binding proteins are no less dynamic, and play important roles in every step of mRNA processing. Indeed, recent screens have uncovered post-transcriptional mechanisms as being integral regulatory mechanisms to neocortical development. Here, we summarize major aspects of neocortical laminar development, emphasizing transcriptional and post-transcriptional mechanisms, with the aim of spurring increased understanding and study of its intricacies.

## Introduction

**Figure 1:**
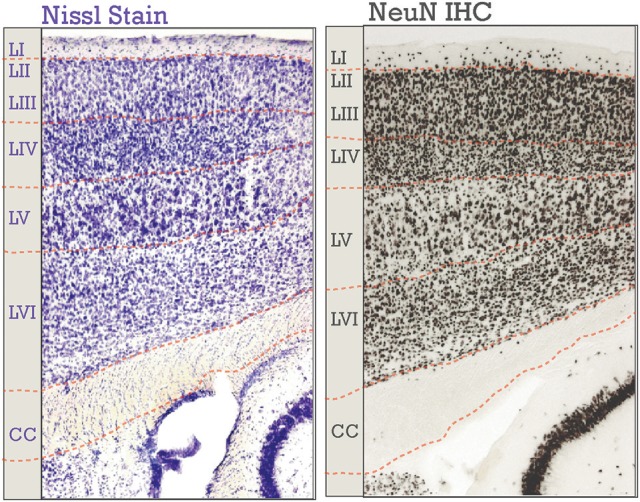
Neocortical six-layered lamination in adult mice. A Nissl stained section is on the left; on the right is an immunohistochemical (IHC) labeling of NeuN. Corresponding boundaries between each layer and the corpus callosum (CC) were drawn in. Layers I-VI are labeled, “LI-LVI.”

The neocortex is laminated brain structure that coordinates our cognitive capacities and responses, long-term memory, sensory and emotional functions, and voluntary motor behavior (Rakic, 2009; Figure 1). More than a century has passed since the organization of the neocortex was identified through by classic neuroscientists including Cajal, Brodmann, Economo, Kskinas, Sarkissov, Bailey, Boning, and others (Douglas and Martin, 2007). The six neocortical layers were first characterized using traditional neuroanatomical techniques, relying on the morphological features of the layers such as thickness, cell density, myelination, and the size of cell perikarya. Modern classification has utilized updated approaches, such as transcriptional profiling and receptor mapping, to identify the layers as unique compartments based on their molecular expression patterns that correspond to classic anatomical boundaries (Molyneaux et al., 2007; Leone et al., 2008; Zilles and Amunts, 2009; Kang et al., 2011; Kwan et al., 2012; DeBoer et al., 2013, 2014; He et al., 2017). The maturation of a stereotyped neocortical structure continues to be an indication of appropriate neuroanatomical development and brain function. Aberrations in neocortical anatomy have been correlated with disease manifestation in autism case studies (DiCicco-Bloom et al., 2006; Amaral et al., 2008; Stoner et al., 2014) as well as in studies of schizophrenia patients (Jones, 1995; Lewis and Levitt, 2002; Wagstyl et al., 2016).

**Figure 2:**
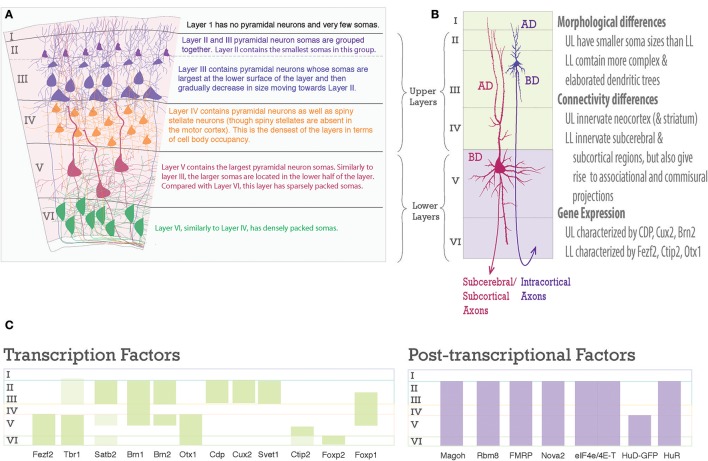
Laminar differences in pyramidal neurons. **(A)** A graphic representation of pyramidal neurons in the six-layered neocortex. To the right are descriptions of the cell body density aspects of each neocortical layer. **(B)** Pyramidal neurons are distinct amongst the upper and lower layers, however, each one shares a common structure. Note the pyramidal somas of Layer III and Layer V neurons, the apical dendrite (AD) extends toward the pia, the basal dendrites (BD) extend toward layer VI, dendrites studded with spines, and an axon projecting toward white matter to go on to their designated targets. On the right is text describing main differences between upper and lower layer neurons. **(C)** Schematic of laminar organization with selected transcription factors, green bars, on the left and post-transcriptional factors, purple bars, on the right. Layers are labeled on left.

Six-layered lamination of the neocortex can be viewed through several different aspects of the neurons contained within. For example, there are the neurons themselves as well as the patterns the projections form. There are two major classes of neurons in the adult neocortex: inhibitory, or Gamma-aminobutyric acid (GABA) utilizing, and excitatory, or glutamate-utilizing. Both classes shows a specific laminar distribution in the neocortex (Jones, 1986; Pla et al., 2006; Molyneaux et al., 2007; Leone et al., 2008; Zilles and Amunts, 2009; Kwan et al., 2012; DeBoer et al., 2013; Tasic et al., 2016), though inhibitory subtypes do not have laminar preference to the same extent as do excitatory neuronal subtypes (He et al., 2017). Pyramidal neurons are glutamate-utilizing projection neurons, represent the majority of neurons (70–85%) in the neocortex (Jones, 1986; Kasthuri et al., 2015), and will be the primary focus of this review (Figure 2). Also of note is that laminar organization is seen in the axonal connectivity of the neocortex, the so-called “myelinated thicket” of projections that traverse the cortex (Jones, 2009). New studies have re-emphasized the importance of myelin organization to brain function (Tomassy et al., 2014; Micheva et al., 2016).

**Figure 3:**
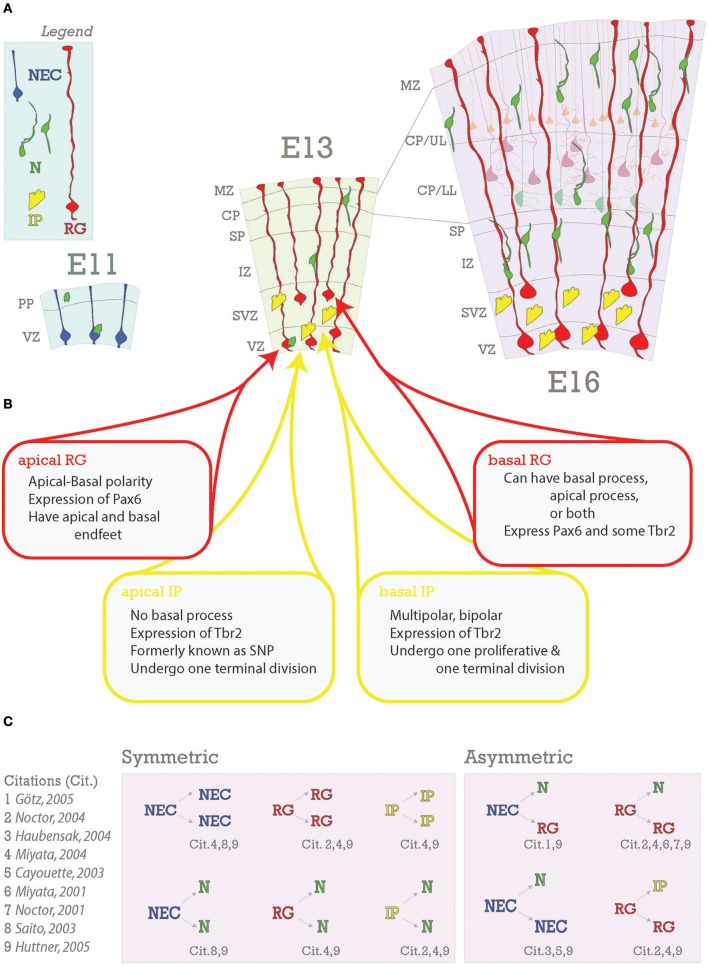
Embryonic foundations of lamination. **(A)** Schematic representation of the progression of neocortical development during prenatal neurogenesis. Legend is in the top left with the main cell types depicted: neuroepithelial cells (NEC), radial glia (RG), intermediate progenitors (IP), and neurons (N). The ages depicted are key stages during prenatal neurogenesis: E11 (onset), E13 (deep layer production), and E16 (transition from deep layer to upper layer and upper layer production). At E11, some neurons have already been generated from NECs and some have arrived from subpallial origins. At E13, pyramidal neurons and progenitors are generated in the VZ from apical RG (aRG) and apical intermediate progenitors (aIPs). In addition, pyramidal neurons are generated in the subventricular zone (SVZ) from basal RG (bRG) and basal IPs (bIPs). Migrating pyramidal neurons pass through the intermediate zone (IZ) and SP to the CP. Later born pyramidal neurons will migrate past the earlier born ones successively to generate the neocortical layers in an inside-out fashion. Nascent layers are seen at E16. Lower layers (LL) have immature neurons and mature neurons already in place (lighter cells in background), extending apical dendrites towards Layer I. **(B)** Heterogeneity of RG and IP progenitors. **(C)** Neurons are generated from both symmetric and asymmetric divisions. References for these events are indicated below each event and detailed to the right.

The layered organization of the adult neocortex stems from the unique placement of its pyramidal neurons, which arise from diverse progenitors during intricate prenatal developmental processes (Figures 2A, 3). Pyramidal neurons are named after the three-dimensional pyramid-like shape of their somata. Pyramidal neurons also have characteristic neurite structure: an apical dendritic tree bearing oblique branches and ending in a terminal tuft, a basal dendritc tree, and a single axon (Jones, 1986; Ramaswamy and Markram, 2015; Figure 2B).

The steps that each pyramidal neuron goes through to form the appropriate layers are **genesis**, **migration**, and **maturation**. While each of these three steps individually has been reviewed extensively (Molyneaux et al., 2007; Kriegstein and Alvarez-Buylla, 2009; Kwan et al., 2012; Shim et al., 2012; Cooper, 2013; Custo Greig et al., 2013; Malatesta and Gotz, 2013; D'Arcangelo, 2014), we will highlight the roles that post-transcriptional regulation plays in these steps (Zilles and Amunts, 2009; Kwan et al., 2012; DeBoer et al., 2013; Pilaz and Silver, 2015; Silver, 2016; Lennox et al., 2017), with a focus on recent literature. Interference at any of these steps can lead to debilitating disorders, such as microcephaly, epilepsy, and cognitive/behavioral impairments (Abdel Razek et al., 2008; Staley, 2015; Willsey and State, 2015; Silbereis et al., 2016; Oaks et al., 2017). For example, recent evidence has uncovered that an epidemic of microcephaly in South America is due to an attack by the Zika virus on neocortical progenitors that give rise to pyramidal neurons (Cugola et al., 2016; Garcez et al., 2016; Tang et al., 2016), affecting the genesis phase of lamination.

Each of the three steps to achieve a laminar six-layered neocortex is guided by transcriptional, post-transcriptional, and epigenetic mechanisms (Leone et al., 2008; Shim et al., 2012; DeBoer et al., 2013; Tuoc et al., 2013b; Pilaz and Silver, 2015; Nguyen et al., 2016). Transcription factors regulate cohorts of genes and as such have been associated with certain subpopulations of cells in the neocortex (Molyneaux et al., 2007; Tasic et al., 2016). Post-transcriptional factors include: RNA-binding proteins (**RBPs**), ribosomal proteins (**RPs**), micro-RNAs (**miRNAs**), and long non-coding RNAs (**lncRNAs**). Recent evidence that will be presented here suggests that these factors also play a crucial role in the generation of the neocortical layers. RBPs are of immense importance. RBPs are not only active in translation promotion and repressing post-transcriptional processing, but also in alternative splicing (AS) and transport/localization of many kinds of RNAs, including the mRNAs encoding transcription factors themselves as well as non-coding regulatory RNAs (Boutz et al., 2007; Chawla et al., 2009; DeBoer et al., 2013; Pilaz and Silver, 2015; Hart and Goff, 2016; Kraushar et al., 2016; Yano et al., 2016; Zheng, 2016). As such, their capacity for intervention in post-transcriptional processing, and consequently all developmental processes, endows cells with an additional layer of regulatory control (Keene, 2007; **Figure 5**).

A thorough understanding of events and molecular mechanisms underlying the development of neocortical lamination will contribute insight to neuronal differentiation and neurodevelopmental/cognitive disorders as varied as microcephaly, autism, and schizophrenia. Here, we review the three stages of neocortical laminar development through exploration of transcriptional and post-transcriptional mechanisms with the aim of spurring increased understanding and future studies.

## Genesis of pyramidal neurons

The laminar destiny of pyramidal neurons is largely established during the embryonic period. Ultimately, each neocortical layer contains neurons that share specific projection patterns, morphological, electrophysiological, and molecular characteristics. These differences are largely genetically programmed according to their prenatal birth date (Molyneaux et al., 2007; Leone et al., 2008; Kwan et al., 2012; Shim et al., 2012; Custo Greig et al., 2013; DeBoer et al., 2013; Harris and Shepherd, 2015; Tasic et al., 2016; He et al., 2017; Figure 2). However, layered subpopulations are often not completely homogenous groups (Arlotta et al., 2005; Molyneaux et al., 2007, 2015; Custo Greig et al., 2013; DeBoer et al., 2014; Sorensen et al., 2015). For example, while lower layer neurons tend to project corticofugally, neurons in layer V have diverse corticocortical and corticofugal axonal projection patterns, including: corticospinal, corticocortical, corticostriatal, and non-specific corticothalamic nuclei in mouse motor cortex (Arlotta et al., 2005; DeBoer et al., 2013; Oswald et al., 2013). Evidence from genetic profiling is in agreement with this characterization: excitatory neurons within a layer are generally more similar to each other than to those in another layer, but still can be grouped into distinct transcriptomic subpopulations. Notably, this is more-so true for lower layers than for upper layers (Tasic et al., 2016). Some studies have gone further and combined retrograde tracing with molecular profiling and found that diverse axonal projections trace back to neurons with distinct transcriptomic patterns (Arlotta et al., 2005; Molyneaux et al., 2007; Custo Greig et al., 2013; Sorensen et al., 2015).

Differences in subpopulations arguably begin to arise from the three main progenitor types present during development: neuroepithelial progenitor cells (NECs), radial glia progenitors (RG), and intermediate progenitors (IPs) (Figure 3A; Gal, 2006; Stancik et al., 2010; Johnson et al., 2015; Pollen et al., 2015; Tyler et al., 2015). These progenitor types overlap in their occurrence; NECs have been prepopulating the nascent ventricular zone when RG first begin to proliferate, RG continue proliferating throughout neurogenesis, and IPs begin to appear after RG and will also continue proliferating throughout neurogenesis. Thus, proportions of progenitors change during the course of development (Noctor et al., 2001, 2004; Götz and Huttner, 2005; Kowalczyk et al., 2009; Pollen et al., 2015; Telley et al., 2016). One tantalizing hypothesis that arises from recent studies is that the diversity of the progenitor pool, changing through the course of development, allows for acquisition of diverse subpopulations in the developed neocortex. The extent to which post-transcriptional regulation plays a role in this process is only recently becoming elucidated.

Pyramidal neurons are born from their progenitors in the dorsal pallium within the proliferative layers named the ventricular zone (VZ) and subventricular zone (SVZ). It is in these zones that neural stem cells divide and begin to generate the cellular diversity of the neocortex. During the first stage of neocorticogenesis, the developing neocortex is composed of just one of these layers- the ventricular zone (VZ) (Figure 3A), which will remain a major proliferative site throughout neocortical development.

### Neuroepithelial progenitor cells (NECs)

The first lineage of neocortical neural progenitor cells, also called neural stem cells and/or neural precursor cells, is composed of NECs. They are identified by Nestin and Sox1 expression (Nestin+ and Sox1+) (Tohyama et al., 1992). Sox1 maintains NECs in their progenitor state (Suter et al., 2009). In the murine neocortex, the earliest neurons will be born between E9 and E10 from NECs and form the preplate (PP) (Bystron et al., 2006); PP cells thus form the first band of neurons above the VZ (**RG are red cells in** Figure 3A; Angevine et al., 1970; Marin-Padilla, 1978; De Carlos and O'Leary, 1992).

**Figure 4:**
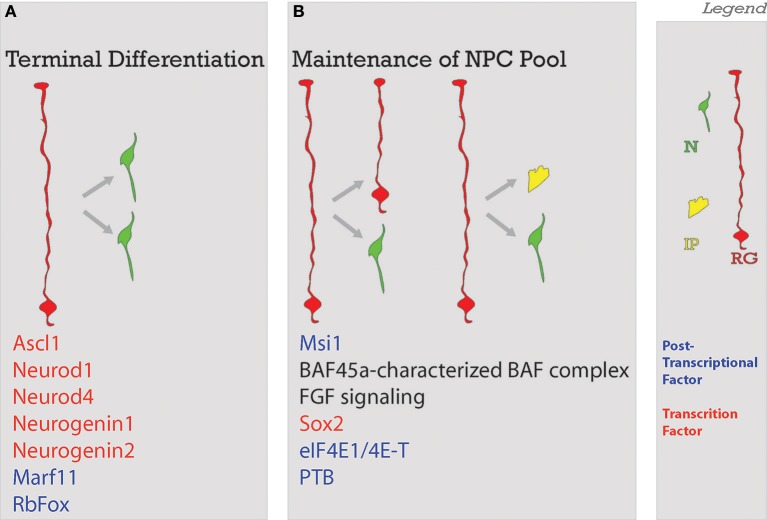
Balance of differentiation and proliferation. This process ensures that an adequate amount of neurons will be produced. Neurons will be produced through **(A)** terminal symmetrical division or in a way that the **(B)** maintenance of the neural progenitor cell pool is ensured with the self-renewal of enough progenitors. Factors guiding each process are listed below. Legend to the right. NSC, neural stem cell; N, neuron; IP, intermediate progenitor; RG, radial glia; Post-transcriptional factors in blue in A and B; TF, transcription factors in red in A and B.

NECs divide symmetrically to produce more NECs (Rakic, 1995; Götz and Huttner, 2005) (Figure 3C), and asymmetrically to generate neurons and RG (Cayouette and Raff, 2003; Haubensak et al., 2004). Symmetric divisions lead either to the expansion of the proliferative pool, resulting in two new stem cells as it is the case with NECs, or to the termination of proliferation with two new neurons or glia (Saito et al., 2003)- frequently seen in later stages of neocorticogenesis (Huttner and Kosodo, 2005). Early divisions of stem cells are important for amplification of the progenitor pool while later divisions tend to be neurogenic (Gao et al., 2014). A depletion of progenitors and increase in neurogenic output during neocorticogenesis has results in decreased cortical thicknesses (Caviness et al., 2003). As a further proof of principle, the *reeler* mutant (discussed in depth in the Migration section of this review), shows disruption in normal balance of proliferative divisions with reduced neuronal production in early stages and increased neuronal production at later stages (Polleux et al., 1998), with the final effect being a severely disorganized neocortex (Guy et al., 2015; Wagener et al., 2016; Guy and Staiger, 2017). As we will demonstrate with the following examples, deficiencies in early neocortical progenitor populations compromise cell fate and localization (Figure 4).

NECs are polarized with apical and basal processes extending over the entire developing neocortex and as such begin to form the structure of the cortex (Kadowaki et al., 2007). They are highly dependent on a stable interaction with the ventricular surface. Apical end-feet, which attach NECs as well as RG to the VZ surface, are regions of cadherin localization and form adherens junctions with the VZ surface to stably attach NECs and RGs there (Kadowaki et al., 2007; Miyamoto et al., 2015). Downregulation of cadherin leads to detachment of these polarized progenitors from the ventricular surface, premature neuronal differentiation, and increased cell cycle exit (Zhang et al., 2010), all leading to a disorganized neocortical structure (Kadowaki et al., 2007). Cadherin localization to apical end feet was found to be dependent on the endocytic adaptor proteins NUMB/NUMBL. NUMB, through N-Cadherin binding, maintains the integrity of the VZ surface and subsequent neocortical organization (Rasin et al., 2007).

The RBP Musashi1 (Msi1) was found to bind mammalian *Numb* (Imai et al., 2001; Yano et al., 2016), and to compete with the translation initiation factor eIF4G to bind poly-A binding protein (PABP). Binding of PABP by Msi1 acts as a translational brake and thereby represses translation of Msi1-bound transcripts, such as *Numb*. Msi1 was found, in retinal Müller glia, to localize to the cytoplasm in mitotic cells and to the nucleus during post-mitotic stages (Nickerson et al., 2011). Thus, in the cytoplasm during mitosis, Msi1 is available to repress translation of Numb and thereby inhibit stable cadherin localization. Numb is decreased in mitotic cells, evidenced by the lack of co-localization with PH3 and P-Vimentin and immunogold electron microscopy (markers of mitosis) (Rasin et al., 2007). It would be fruitful to confirm that this pattern of Msi1 subcellular localization holds true in cortical NECs/RG. This would further demonstrate that Msi1 indeed can act in a cell-cycle-locked manner to promote neocortical stem-cell proliferation through regulation of Numb.

Notably, this is just one mechanism by which Msi1 maintains stem-cell fate. The most prominent example, regulation of Notch through Numb, has been thoroughly elucidated by Okano and others (Imai et al., 2001; Kohyama et al., 2005). Recent evidence has also implicated Msi1 as necessary for ZIKV replication (Chavali et al., 2017), paradoxically leading to the death of the very cells Msi1 normally maintains. Dependence on the RBP Msi1 further demonstrates why ZIKV is so efficient in its targeting of neural progenitor cells and subsequent formation of the tragic microcephaly phenotype.

Interestingly, knockouts (KO) of a nuclear protein Akirin2 show a phenotype that is similar to, but more drastic than, cadherin disruption. Akirin2 KOs show increased cell cycle exit, disorganized neocortex, and down regulation of N-cadherin and Connexin-42 protein. Downregulation of these genes leads to a loss of adherens junctions and a “spilling” of progenitor cells into the lateral ventricle (Bosch et al., 2016). Akirin2 acts as a bridge between transcription factors and BAF complexes, which are epigenetic chromatin remodeling complexes (Bosch et al., 2016). Called SWI/SNF in yeast and BAP in *Drosophila*, the BAF complex is composed of ten proteins which have been shown to differentially associate in specific cell types. The BAF45a/53a subunits are highly expressed in progenitors and BAF45a is sufficient to keep progenitors in their proliferative state. When a cell becomes post-mitotic, there is a repression of BAF45a/53b and a switch to expression of BAF45b/45c/53b (Lessard et al., 2007). While loss of individual BAF subunits results in expression changes in specific genes, total loss of these complexes leads to global effects on gene expression. This was demonstrated with the generation of the BAF155/170 double conditional KO- in these mutants no BAF complexes form. Researchers determined that there was a concomitant increase in heterochromatin formation due to increased H2K27Me2/3 methylation in the telencephalon (Narayanan et al., 2015; Nguyen et al., 2016). Together, these experiments demonstrate the local and global roles that tight epigenetic control can have over cell fate.

Another RBP, Hu antigen R (HuR), could play a role in maintaining the appropriate balance of NECs to neurons. HuR was found to be expressed in a NEC-like cell line (H2-b2T) at high levels during mitosis (M-phase), during growth (G1 and G2), and at low levels during synthesis (S-phase) (Garcia-Dominguez et al., 2011). HuR stabilizes mRNA transcripts through binding of AU-rich elements (AREs); though notably AREs were initially identified to lead of instability of transcripts (Fan and Steitz, 1998; Peng et al., 1998; Brennan and Steitz, 2001). During M-phase, HuR stabilizes *Delta-like 1* (*Dll1*) mRNA via interaction with ARE-elements in *Dll1*. Stabilization likely allows for high levels of *Dll1* to be obtained, which in turn allows the NEC expressing *Dll1* to differentiate while laterally inhibiting its neighbors from differentiating (Louvi and Artavanis-Tsakonas, 2006). Briefly, neighbors of the *Dll1*-high cell are expressing Notch and upon interaction with the ligand, transcription of neuronal-fate repressors (bHLH genes *Hes1* and *Hes4*) is enacted (Ohtsuka et al., 2001; Liao and Oates, 2017). Either decreased DLL1 or decreased Notch means that pro-neuronal genes will be transcribed (Appel et al., 2001; Homem et al., 2015). HuR heterozygotes in this study were shown to have a decreased expression of *DlI1* at E10.5 (Garcia-Dominguez et al., 2011). By postnatal day 0 (P0), *HuR* conditional knockout, with deletions at the NEC stage (*Foxg1-Cre*) and the RG stage (*Emx1-Cre*), have significantly reduced cortical thicknesses (Kraushar et al., 2014).

### Radial glia

The first pyramidal neurons are born about a day later around E11.5 and migrate toward the pial/basal surface to form the cortical plate (CP). These CP neurons split the PP into the superficial marginal zone (MZ) and the deeper subplate (SP) (Molliver et al., 1973; Kostovic and Rakic, 1990; Allendoerfer, 1994). From there, each successive group of neurons will migrate past those already present to form nascent layers. As neurogenesis progresses, there is an increase in the expression of Tbr2, a marker of intermediate progenitors (IPs) in the SVZ (IPs are yellow cells in Figure 3A; Englund, 2005). Once the bulk of embryonic neurogenesis ends at E18, RG will give rise to glial lineages. In the adult brain, neurons are accompanied by astrocytes, microglia, oligodendrocyte precursor cells, oligodendrocytes, and endothelial cells (Rakic, 1995, 2009; Molyneaux et al., 2007; Leone et al., 2008; Kwan et al., 2012; DeBoer et al., 2013; Tasic et al., 2016).

The genesis of pyramidal neurons is characterized by the switch from NEC-characteristic symmetric divisions to RG-characteristic asymmetric ones (Götz and Huttner, 2005). RG are a more specialized lineage of neural stem cells and have both basal and apical processes spanning the extent of the length of the nascent neocortex, similar to NECs. However, in contrast to NECs, RG asymmetric divisions result in two different kinds of daughter cells: one self-renewing RG and one either terminal neuron, IP, or RG (Noctor et al., 2004, 2008).

This important switch from NECs to RG is regulated by both extrinsic and intrinsic factors. The extracellular factor Fgf10 in the rostral, but not caudal, part of the neocortex favors a rapid transition to RG fate (Sahara and O'Leary, 2009). Experiments involving the deletion of cortically-expressed FGF receptors (−*1*, −*2*, and −*3*), demonstrates that FGF signaling maintains RG in a proliferative state (Kang et al., 2009). Intrinsically, Pax6 expression drives NECs to RG fate (Suter et al., 2009). Sox2 has been identified as a maker of RG (Hutton and Pevny, 2011), but is also expressed at low levels in IPs (Pollen et al., 2015). Unlike NECs, RG are not restricted to the VZ; those that stay at the VZ are called **aRG** and those that are found in the SVZ are called **bRG**, also called outer RG (oRG) (Miyata et al., 2004; Noctor et al., 2004, 2008; Götz and Huttner, 2005; Huttner and Kosodo, 2005; Kowalczyk et al., 2009; Kriegstein and Alvarez-Buylla, 2009; Hansen et al., 2010; Wang et al., 2011).

aRG express PAX6, several astroglial markers (e.g., GLAST and BLBP), and maintain apical-basal polarity (Hutton and Pevny, 2011). bRG were recently split into three subtypes: unipolar with a basal process attached to basal lamina, unipolar with an apical process attached to the pial surface, and bipolar. bRG all express PAX6, but many also express Tbr2 (Betizeau et al., 2013). Recently, a screen aiming to transcriptionally profile the outer SVZ (OSVZ), found that while cells that expressed *Tbr2* indeed had diverse morphological characteristics, they had transcriptomes distinct from classic RG molecular profiles (Pollen et al., 2015). *In vitro* experiments have demonstrated that all TBR2-expressing cells have once had RG markers, but progressively go through “transcriptional waves” rather than drastically shift in their expression patterns (Telley et al., 2016). With the plethora of new molecules that can be used for subtype identification in these screens, enhanced identification and distinction amongst progenitors is on the horizon (Figure 4B).

There is a proven dependence of early-born progenitors on cell-cycle stage in their fate decision (McConnell and Kaznowski, 1991). Using auto-radiographic tracing with [^3^H] thymidine, ferret RG from E29 (when deep layers are being generated in ferret) were heterochronically (“different time”) transplanted into postnatal ferrets. 24 h after transplantation, >85% of migrating cells were found in layer VI. If cells were transplanted immediately after being labeled (still in S-phase and thus able to incorporate the label), ~85% of them migrated to layers II/III. Proliferative cells in the murine VZ have also been found to stay clustered with their “sisters” (Cai et al., 1997). Finally, of the cells that continued to divide in the ferret host cortex (Identified by a diluted [^3^H]thymidine), 98.3% migrated to layer II/III. These results suggest that the environment in which the RG cycles can provoke the RG to acquire a certain fate. Yet, if the RG it has gone through its final S-phase, it seems to have made its decision and its terminal daughter cell will follow this instruction (McConnell and Kaznowski, 1991).

Recent evidence demonstrates that progenitor cells going through a self-renewing cell-cycle will have longer S-phases than those producing a neuron (Arai et al., 2011), suggesting that the window of opportunity to make a fate decision is increased. Indeed, this was likely observed with clonal analysis on clusters of proliferative cells (Cai et al., 1997) as well as demonstrated by manipulating a cell-cycle inhibitor (p27) to increase cell cycle exit (Caviness et al., 2003). This is further reflected in KOs of Dmrta2, a pro-neuroepithelial gene, where more cells in the knockout are in G0/1 (Young et al., 2017) and fewer in S compared to controls (Konno et al., 2012; Young et al., 2017). This suggests increased cell cycle exit; indeed, forced expressed of the pro-neuroepithelium factor *Dmrta2* leads to an increase in Ki67+ cells (Young et al., 2017).

**Figure 5:**
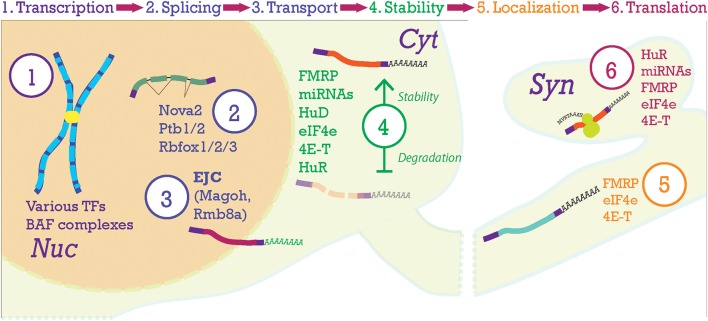
Post-transcriptional processing steps in the neocortex. At the top of the figure, the progression of mRNA post-transcriptional processing is shown. **Transcription** begins in the nucleus (Nuc) from chromosomes producing pre-mRNA. Pre-mRNA is processed in the nucleus where the Exon-Junction Complex (EJC) will label the mRNA after **splicing** and help with nucleo-cytoplasmic **transport**. Once the transcript is moving to and throughout the cytoplasm (Cyt), **stability** of the mRNA becomes paramount. Here, transcripts will be stabilized (top transcript) or degraded (lower transcript). Then, transcripts will be **localized** to their final compartments of **translation**, in this case the synapse (Syn).

Acting through post-transcriptional means, specific translational partners have been found to directly contribute to neurogenesis. Yang et al. identified eIF4E, eukaryotic initiation factor 4E, and 4E-T are interacting protein partners. Using immunohistochemistry, they found that 4E-T colocalized with eIF4E1 70% of the time; though eIF4E1 colocalized with 4E-T only 2.7% of the time (Yang et al., 2014)- possibly pointing to other interacting partners like 4E-T for translation of specific transcripts. The sites of colocalization were in Processing-bodies (P-bodies) (Yang et al., 2014). P-bodies are a kind of ribonuceloprotein (RNP) complex, similar to, but distinct from stress granules (Kedersha et al., 2005; Decker and Parker, 2012). These are hypothesized to be packages of inactive translational machinery which are important for local translation (Decker and Parker, 2012; Figure 5, step 5). In an elegant series of experiments, the authors demonstrated that when protein of Neurogenin1 or Neurogenin2 was present, the mRNA transcripts of either were not colocalized with 4E-T+ RNPs. Both eIF4E1 and 4E-T were found to maintain cells in a progenitor state and repress neuronal fate by directly repressing neuron-fate promoting transcripts *ascl1, neurod1, neurod4, neurogenin1*, and *neurogenin2* from translation (Yang et al., 2014). It should be noted that a direct interaction between 4E-T and mRNA has been demonstrated, suggesting that 4E-T requires a capable partner to enact this translational repression. Importantly, eIF4E1 knockdown experiments resulted in precocious differentiation, not increased cell death. This demonstrates that the phenotype seen here is not simply a consequence of wide-spread translational deficiency, but rather the result of interference in a fate-determining pathway.

More subtly, experiments with eIF4E1/4E-T demonstrated that the mere presence of mRNA (i.e., *neurod4*) should not be correlated with protein expression- as active repressive mechanisms operate post-transcriptionally to control transcript translation. This pattern is seen repeatedly in RBP-control of mRNAs (DeBoer et al., 2013, 2014; Kraushar et al., 2014, 2016; Popovitchenko et al., 2016). Normal mRNA expression and absent protein is a hallmark of post-transcriptional regulation.

Recent studies have shown an expansion in the types of neuronal progenitors through evolution (Florio et al., 2015; Johnson et al., 2015; Pollen et al., 2015). Specifically, an expansion of the SVZ has been found in primates such that the SVZ is divided into outer (oSVZ) and inner (iSVZ) portions. Johnson et al. used fluorescence-activated cell sorting (FACS) to separate cell types in the developing human brain in order to understand transcriptional programs in human neural progenitors (Johnson et al., 2015). Three groups were isolated based on expression of cell-surface markers LeX, GLAST, and high/low/negative Prominin: (1) aRG (similar to typical rodent RG), (2) IPs and neurons, and (3) non-aRG called outer radial glia (oRG, an expanded cell type in primates present in very few numbers in rodent (Wang et al., 2011). Relying on combinations of cell-surface markers in sorting ensures an enrichment of a desired subpopulation, which can be advantageous of the population of interest is scarce. As with all methods, there are drawbacks, as it should be noted that the use of LeX (also called CD15) and Prominin (also called CD133) as markers of NPCs has been shown to be exclusive of at least one highly-proliferative population of cells (Sun et al., 2009; Hutton and Pevny, 2011). Despite this, the combined power of the single-cell sorting achieved with FACs and the unbiased profiling with RNA-seq allowed for novel discovery, specifically of post-transcriptional mechanisms responsible for isoform differences and non-protein coding elements- aspects of the transcriptome that other methods like a microarray would not allow for.

Johnson et al. uncovered that long non-coding RNAs (lncRNAs) (Hart and Goff, 2016) account for at least a part of the diversity of progenitors between higher-order mammals and rodents (Johnson et al., 2015). Specifically, 253 unannotated human-specific loci were identified; 2.4% specific to ORG and thus, given that they were unannotated, were postulated to be lncRNAs (Johnson et al., 2015). This is particularly intriguing as lncRNAs were found to be expressed at relatively low levels in the brain, and hypothesized to have high cell-type specificity (Cabili et al., 2011)- possibly in different progenitor types. That lncRNAs have lower levels of gene expression than protein-coding genes was confirmed in another RNAseq screen of mouse cortical excitatory neurons at all developmental ages examined (Molyneaux et al., 2015). During development, Molyneaux, Goff et al. found that ~56% of lncRNAs are associated with “cell-type specific clusters” of neurons, while ~32% of transcripts associate with “cell-type independent” clusters, i.e., not defined by high individual levels of *Bcl11b, Satb2*, or *Tle4* expression (Molyneaux et al., 2015).

Another study mapped seventy-six cortical lncRNAs (specifically: *intergenic* non-coding RNAs, “lincRNA,” long, > 200 basepairs), and found them to be layer-specific (Belgard et al., 2011). These findings suggest that non-protein coding transcripts like lncRNAs can substantially impact lamination and vice-versa. Furthermore, a specific lncRNA, lncND, has been associated with intellectual disability (ID), and was found to be a part of an ID-associated microdeletion in six affected individuals (Rani et al., 2016). To further explore the extent of the contribution of lncRNAs to neurodevelopment, D'Haene et al. carried out a transcriptomic screen enriched for lncRNAs in human models. Researchers found 53 lncRNAs with high correlation to intellectual disability and that contained a disease-associated SNP (D'Haene et al., 2016). Further confirmation of these results in murine models would aid in the development of tractable models of ID.

Overall, we can see that there is a diverse regulatory machinery which we may be able to tap for more specific distinction amongst progenitor types, and that post-transcriptional processing plays a large role in establishing this diversity and ultimately a high-functioning cortex.

### Intermediate progenitors

Appropriate production of progenitors is the result of the balance between proliferation and neurogenesis during development. IPs are posited as the major intermediary neurogenic source. Several transcriptional and post-transcriptional mechanisms are involved in the adoption of IP identity.

IPs arise as a result of *Pax6* downregulation and *Tbr2* upregulation in these cells (Martynoga et al., 2012). Seemingly paradoxically, the onset of Tbr2 expression is in fact positively regulated by Pax6 binding (Sansom et al., 2009). Though the *Tbr2* transcript is produced, *miR-92a* has been found to maintain the RG pool by targeting *Tbr2* for translational repression (Figure 5, step 4); co-electroporation with a Tb2-protector that blocks binding of the miRNA to the Tbr2-3′ untranslated region (UTR) allowed for accumulation of Tbr2 protein and transition to IP fate. *miR-17-92* conditional knockout experiments show a decrease in RG and a concomitant increase in IPs (Bian et al., 2013). Authors demonstrated expression of the miRNA cluster in the proliferative zones, but did not identify the specific progenitors expressing it. Studies examining *miR-92b* showed similar results, though did additionally localize higher expression of the miRNA in the VZ and lower expression in the SVZ, with varying levels amongst cells (Nowakowski et al., 2013). Though another group has shown that expression of *miR-92b* decreases as Tbr2 increases (Nielsen et al., 2009), it remains unclear what mechanism allows for increased Tbr2 expression in IPs. Three possibilities (of many) are that the miRNA itself is targeted, it is no longer transcribed, or that some factor protects *Tbr2* from the miRNAs. This protector could very well be an RBP as competition between RBPs and miRNAs is a common theme in stable mRNA expression (Gardiner et al., 2015). As an aside, specific interaction between lncRNAs and miRNAs has also been demonstrated (Rani et al., 2016), further expanding the competitive dynamics of the post-transcriptional regulatory framework.

Epigenetic factors also contribute to the switch from RG to IP. Two BAF complex members, BAF170 and BAF155, switch in different fates and compete to interact with Pax6. BAF170, normally expressed at higher levels in more differentiated cells, is an intrinsic factor of RG and the conditional knockouts shows enhanced production of Tbr2+ IPs. This is due to increased incorporation of BAF155, normally expressed at higher levels in less-differentiated cells (Tuoc et al., 2013a,b). Considering the neurogenesis-specific genes that Pax6 regulates (Cux1, Tle4, Tbr2), timely access to its targets through “loose” euchromatin ensures appropriate laminar development (Figure 5, step 1).

Similarly to RG, IPs come in two varieties in the neocortex: apical and basal (Figure 3A). Apical intermediate progenitors (aIPs) were identified along with aRG in the VZ during neurogenesis (Englund, 2005; Tyler and Haydar, 2013). Unlike RG, IPs do not have a basal process and downregulate expression of astroglial markers. Additionally, instead of self-renewal stages of division, aIPs undergo terminal symmetric division to produce two neurons, amplifying the neuronal output. bIPs completely lose astroglial markers and Pax6 expression, but they gain Tbr2 expression in the SVZ (Englund, 2005). bIPs first divide symmetrically to produce two more bIPs and then once again symmetrically, the result of which is four neurons. Therefore, these bIPs are also called “transient amplifying cells” (Noctor et al., 2004; Stenzel et al., 2014) or “transient amplifying progenitors” (Betizeau et al., 2013).

Tbr2 was first identified as an important factor in the generation of upper layers and, is expressed in all bIPs and to an extent in bRG. In a conditional Tbr2 knockout, lower layers form normally and the majority of cells in upper layers are also distributed normally (Sessa et al., 2008). However, the subpopulations expressing the transcription factors Satb2 and Brn2 are reduced, implicating Tbr2 as a crucial upstream transcriptional step in subpopulation-specific neuronal development. Though upper layers in Tbr2 conditional knockouts are present and several examined subpopulations are unaffected, mice display increased aggressiveness and participate in infanticide (Arnold et al., 2008). This demonstrates that imbalances in Tbr2-derived subpopulations can yield severe cognitive deficits, while having no gross effects on lamination.

Several lines of evidence demonstrate that lower layers are also populated by neurons from the Tbr2+ lineage (Englund, 2005; Sessa et al., 2008; Kowalczyk et al., 2009; Mihalas et al., 2016). In a recent study, Tbr2+ cell fate on a given day was found to be similar to overall cell fate that day; i.e., neurons born early from IPs will be found in lowers layers (Ctip2+) and neurons born later from IPs will occupy upper layers. A portion of early-born Tbr2+ cells continue proliferating, as ~14% of cells generated at E13.5 and ~68% at E16.5 ended up in upper layers (Mihalas et al., 2016). It would be interesting to further evaluate if Tbr2 is sufficient to maintain the proliferative ability of a cell. Conditional knockout of Tbr2 resulted in an increase of early-born lower layer neurons (Mihalas et al., 2016), suggesting an exit from the cell-cycle and early termination of the stem-cell program. Authors conclude that Tbr2 is necessary for proper neuronal differentiation as opposed to the genesis of IPs (Mihalas et al., 2016).

Tbr2 has been useful in distinguishing progenitor types and more importantly in understanding how different progenitors can build the myriad of subpopulations in the laminar neocortex. One study in particular has elegantly demonstrated how progenitor heterogeneity directly leads to diverse laminar subpopulations. Tyler et al. found that they could identify two distinct post-mitotic populations in the same cortical layer based on the presence (Tbr2+) or absence (Tbr2-) of Tbr2. These cells were shown to be born at the same time, but had unique identities. The populations arising from the SVZ, Tbr2+ cells, had less complex branching than cells originating from progenitors in the VZ, mostly Tbr2-, and a higher input resistance corresponding to higher excitability (Tyler et al., 2015). These results demonstrate that Tbr2 expression in a progenitor will lead to a different downstream neuronal identity and transcriptional program than those cells which do not express Tbr2 (Stancik et al., 2010).

A recent screen also isolated NPCs based on their gene expression of Tbr2. Tbr2- cells were confirmed as being Sox2+, and were thus called the RG-NPC population. Tbr2+ cells are IPs and early postmitotic neurons (Englund, 2005; Hutton and Pevny, 2011). Cells were processed with FACS and RNA-seq. The screen revealed that alternative splicing regulated 622 exons differentially between Tbr2- NPCs and Tbr2+ cells (Zhang et al., 2016). The most common regulatory event (accounting for ~37% of alternative splicing events) was a skipped exon. 61% of skipped exons contained regulatory motifs of two prominent splicing factors, Ptb1/2 and/or Rbfox1/2/3 (Figure 5, step 2). This further highlights the immense regulatory potential and role of RBPs in both maintaining progenitor populations and transitioning to neurogenesis (Zhang et al., 2016). Ptb1 was previously implicated in actively repressing neuronal differentiation and conversely Rbfox was previously implicated promoting neuronal identity (Boutz et al., 2007; Gehman et al., 2011; Xue et al., 2013). The Zhang et al. screen of NPCs corroborates both of these roles and provides further mechanistic information. Authors of the screen showed that Ptb1 is a potential target of Sox2 in NPCs and that Rbfox overexpression resulted in fewer progenitors suggesting a premature switch to neuronal fate (Zhang et al., 2016). Alternative splicing thus emerges as one of the post-transcriptional mechanisms responsible for generating a heterogeneous progenitor pool (Figure 3B).

Efficient alternative splicing requires the actions of the spliceosome. After splicing, the spliceosome labels mRNA transcripts with a group of proteins called the exon-exon junction complex (EJC) (Figure 5, step 3). The EJC is usually located 20–24 nucleotides upstream of an exon-exon junction. It has been found to be required for the efficient splicing of some introns, but not all (Fukumura et al., 2016). Its presence has also been found to promote transport out of the nucleus as well as provide an anchoring point for NMD-proteins Upf2 and Upf3 (Le Hir, 2001; Hir et al., 2015). A mutagenesis screen identified one of the members of the EJC complex, Magoh, as responsible for a microcephaly phenotype. Upon investigation of the small brains from Magoh haploinsufficient mutants (*Magoh*^*Mos*2/+^), the group found a depletion of Tbr2+ IPs, but not Pax6+ RG, from E13.5-E16.5 with a concomitant decrease in dividing IPs. Interestingly, Cux1+ upper layers were reduced and disorganized in the Magoh mutant (Silver et al., 2010). Another member of the EJC, Rbm8a, was also found to cause defects in lamination and microcephaly. In addition to a decrease in Tbr2+ IPs, Rbm8a haploinsufficient mutants also had a decrease in Pax6+ RG by E13.5. Concommitant with a decrease in progenitors, an increase in neurons was seen at early stages. By postnatal day 0, Cux1 (layer II/III) and Foxp1 (layers III-V) subpopulations, but not Tbr1 (layer VI), were essentially depleted (Mao et al., 2015).

The mechanism behind progenitor deficiencies in Magoh haploinsufficient mutants was further explored and found to be related to the cell cycle length. Using live imaging, authors found that the prometaphase and mitosis stages of the cell cycle in mutant RGs was increased by 2.4-fold. Magoh mutant progenitors also underwent more neurogenic divisions at E12.5 and had more apoptotic neurons. Finally, using pharmacological inhibitors of mitosis, authors recapitulated the phenotypes seen with live-imaging; namely, that more neurons were produced and 25% fewer IPs were produced when the cell cycle was lengthened (Pilaz et al., 2016b).

The RNA-binding protein (RBP) Marf1 was found to contribute to the generation of Tbr2+ cells and the reduction of Pax6+ cells. Overexpression of the somatic form of Marf1 at E13.5 resulted in increased Satb2+ neurons (corticocortical projection neurons) at postnatal day 2, though did not impact overall lamination, suggesting premature terminal differentiation. Marf1 was found to function through a somewhat unique mechanism: repression of transcripts through its RNAse activity (Kanemitsu et al., 2017), further demonstrating the wide range of activity that RBPs can have.

The RBP Fragile-X Mental Retardation Protein (FMRP) has also been implicated in regulating the RG to IP transition in neurons. It was first posited that FMRP interacts with cytoskeleton proteins in RG (Saffary and Xie, 2011). One mechanism that supports this hypothesis was recently proposed by Debra Silver's group. In RG, FMRP acts as an active transport vehicle for endfoot-localized mRNA and authors effectively used live-imaging to demonstrate this. By following EGFP-FMRP in organotypic slice cultures, they found that the protein moves to localize in RG basal endfeet. Using RIP-chip, the FMRP-associated basal endfoot transcriptome was identified. Importantly, 31% of transcripts isolated from RG basal endfeet were associated with neurological disease. With the use of the photoconvertible molecule Dendra2 and the translation inhibitor anisomycin, the authors determined that these transcripts were being actively transported into basal endfeet (Pilaz et al., 2016a).

## Migration

The day of birth of a pyramidal neuron from progenitors determines its location, and ultimately its identity and function (Pollen et al., 2015; Tasic et al., 2016; Telley et al., 2016; He et al., 2017). The earliest-born neurons take their place in layer VI and the next wave of neurons will form layer V; both groups project subcortically. Later-born neurons will give rise to intracortically projecting neurons mainly occupying layers II-IV, but can also be found in deep layers at lower numbers. In this way, the neocortex is formed in an “inside-out” fashion, with the deepest neurons born first and the most superficial ones born last.

The generation of neocortical layers has been faithfully reproduced using several methods in mice and higher mammals. The landmark study by Angevine and Sidman in laminar cortical development first demonstrated that progenitors labeled at a certain stage are destined for a certain population by postnatal day 10 (P10, late in development) in mice. This is most apparent when progenitors and their progeny were observed at E17; only neurons close to the pial surface are labeled by thymidine showing that active progenitors at this time point will give rise to progeny in upper layers (Angevine and Sidman, 1961).

In another series of classic experiments, McConnell et al. demonstrated that the fate of progenitors is, again, mostly predictable based on their birthdate. Isochronic transplantation (isochonronic: “same time”) experiments were conducted in ferrets during P1 and P2, when upper layers were being generated in this animal (this neurogenic period corresponds to ~E14.5-E18 in rodents). VZ tissue, which contains RG, from donor ferrets that were previously injected with tritiated-thymidine ([^3^H]thymidine) was dissected out, cells dissociated, labeled, and injected into the ventricular zone of a same-age host ferret. Hosts were sacrificed at several timepoints after transplantation in order to observe long-term and immediate cell migration. By two months, the long-term period, ~90% of injected cells had taken their positions in layers II/III (McConnell, 1985).

Modern approaches have further confirmed these classic experiments. Observing sparse labeling in mosaic mice, investigators have been able to begin to determine the relative contributions of progenitors in Mosaic Analysis with Double Markers (MADM) mice (Tasic et al., 2012; Hippenmeyer et al., 2013). NECs/RG labeled at an early age (E10) will give rise, eventually, to neurons spanning the upper layers II-IV (~55%) and lower layers V-VI (~45%). In keeping with the birthday being predictive of laminar location, progenitors actively dividing later in neurogenesis (E15) overwhelmingly give rise to neurons that will occupy layers II-IV (98%) (Gao et al., 2014). More recently, using a FlashTag (FT) approach, similar results were demonstrated. FT, similarly to [^3^H]thymidine, dilutes upon division so progeny are easy to trace. Laminar fate was tightly locked to birthday so authors were able to trace cells born at E14.5 that would eventually take their place in layer IV; authors also demonstrated that cells generated at E14.5 could remarkably be distinguished from cells generated at E14.0. Using this precise temporal pattern to their advantage, authors dissected and sorted cells at 6, 12, 24, and 48 h post-mitosis using FACS. Transcriptomes of cells demonstrated distinct “waves” of gene expression that correlated with the biology occurring at those time points. For example, at the 6 and 12 h time points, proliferation-associated genes were downregulated and translational factors were increased (Telley et al., 2016).

The *reeler* mouse has a null version of the Reelin protein and disorganized cortical layers. It is necessary for the proper migration of cortical neurons. It has also been observed to affect radial glia scaffolds (D'Arcangelo, 2014), which immature cortical neurons utilize in migration, as well as glia-independent somal translocation (Miyata et al., 2001; Franco et al., 2011). Reelin, a factor secreted by Cajal-Retzius cells, acts through the receptors ApoER2 and Vldlr. These receptors bind a central fragment of cleaved reelin, but not the N- or C- termini. Both phosphorylate Dab1, the downstream effector of reelin signaling. Mutations in all of these reelin-associated proteins result in laminar disorganization, though the reelin mutation itself is the most severe suggesting undiscovered redundancy in the pathway (D'Arcangelo, 2014). The extent of laminar disorganization seems to be dependent upon cortical area (Polleux et al., 1998). Paradoxically, in studies of mutant reeler mice connectivity and postnatal functionality are largely preserved (Polleux et al., 1998; Guy et al., 2015).

Notch turns out to be involved not only in genesis of neurons, but also in control of their lamination through Reelin signaling. When examining *reeler* mice, researchers observed that while full-length Notch1 was not different between *reeler* and control mice, the intracellular domain (NICD) of Notch1 was not present in nuclei of *reeler* mice (Hashimoto-Torii et al., 2008). The NICD is the enactor of the Notch transcriptional pathway such that its absence predicts precocious neuronal entry. To test the significance of a mislocalized NICD, Cre recombinase was electroporated along with a fluorescent reporter into an E14.5 floxed double mutant cortex. Over 50% of cells ended up beneath Layer VI, while electroporation in control heterozygous cortices resulted in ~90% of neurons being localized to layers II-IV; interestingly suggesting a failure to migrate in the Notch mutants. This mechanism was found to be dependent on Dab1-Notch1 interaction and specific to post-mitotic neurons (Hashimoto-Torii et al., 2008).

The Nova2 RBP knockout mouse has a disorganized laminar phenotype reminiscent of the *reeler* mouse (Yano et al., 2010). Some, but not all, neurons expressing upper layer markers were incapable of migrating past layer V. It was found that divisions at E14, but not E12, were responsible for mislocalized neurons; consistent with the observation that some upper layer cells were unable to migrate but lower layer neurons were relatively normally-positioned. It was demonstrated that Nova2 regulates Dab1 through inhibition of exon inclusion and seems to be important for late-born neuronal migration (Yano et al., 2010). Thus, Nova2 is an example of a post-transcriptional regulator, and specifically of an RBP, that can affect migration. Its regulation of the reelin pathway is through exon exclusion, a kind of AS (Matera and Wang, 2014).

The odyssey of neurons from the VZ to the CP have been grouped into four stages: (1) cells in the VZ take on a bipolar morphology and migrate toward the SVZ, (2) migratory arrest and assumption of a multipolar morphology, (3) migration *back* toward the VZ and again a bipolar morphology is observed, and (4) final phase in which cells migrate from the VZ to the CP and also reverse their polarity (Noctor et al., 2004; Tan and Shi, 2013). Phases of migration are thus found to correlate with morphology. In the knockout of Fmr1, neurons do not always migrate to their appropriate layers; though major aspects of cytoarchitectonics are retained. The multipolar to bipolar morphology transition during the migration steps was found to be defective in this mutant, suggesting that adoption of bipolar morphology is important for laminar positioning. N-Cadherin2 (*Cdh2*) regulation by FMRP was found to be responsible for this transition (La Fata et al., 2014). A separate study identified the gap junction proteins Connexin-26 and Connexin-43 as necessary for RG-guided migration of neurons (Elias et al., 2007). Molecules such as Notch and gap-junction proteins function differently during genesis and migration because of the different cell types they are acting in and consequent to what regulatory factors are active at the time.

## Postmitotic postmigratory maturation

Once a neuron has migrated past older cohorts of neurons and reached its target destination in the cortical plate, it begins to transition to final maturity. This process begins in the prenatal period and continues well into the postnatal period. The period of extended maturation is most dramatic in primates, where laminar signatures begin to show signs of maturation at around 1 year old (Lein et al., 2017). Intriguingly, *in vitro* preparations of cortical neurons recapitulate aspects of development, such as gene progression (Telley et al., 2016) and laminar markers (Handel et al., 2016). While a neuronal stem cell can feasibly make a mature group of neurons (Gaspard et al., 2008), it will ultimately be a confluence of both extrinsic effectors (Kraushar et al., 2015) as well as continued intrinsic maturation driven by transcriptional as well as post-transcriptional mechanisms that generate a functional neocortex as characterized by **gene expression** and **circuit integration**.

There are several classifications of cortical pyramidal neurons based on their connectivity, otherwise known as hodological classification. Neocortical pyramidal neurons have been grouped into callosal projection neurons, layer IV granular neurons, forward and backward projection neurons, corticostriatal, corticothalamic, subcerebral, and corticospinal motor neurons (Custo Greig et al., 2013). Another classification more succinctly divides pyramidal neurons into three main categories based on these diverse projection types: **intratelecephalic (IT)** with axons projecting to neocortex, striatum, amygdala, claustrum; **pyramidal tract (PT)** with axons projecting to subcerebral targets, i.e., brainstem, spinal cord, and midbrain; and **corticothalamic (CT)** with axons projecting to the ipsilateral thalamus (Harris and Shepherd, 2015). It should be noted that like excitatory neurons, inhibitory neurons also establish circuits with non-cortical brain regions as well as within the cortex (Tamamaki and Tomioka, 2010; Lee et al., 2014; Tomioka et al., 2015). Broadly, we can understand the framework of the neocortex by dividing it into its upper and lower layers (Figure 2B). Neurons in the upper layers project to their own cortical hemisphere (associative neurons) and/or to neurons to the neighboring hemisphere (commissural neurons). In addition to some commissural and associative neurons, lower layers have more diverse targets located subcortically (thalamus, amygdala, claustrum- IT and CT neurons) and subcerebrally (midbrain, spinal cord- PT neurons (Molyneaux et al., 2007; Feldmeyer, 2012). Thus, the three categories mentioned- IT, PT, and CT- comingle in the lower layers but not in the upper layers, which are primarily IT neurons (Harris and Shepherd, 2015).

Development of appropriate axonal and dendritic projections is a key event in neuronal maturation. In a classic transcriptomic screen for cortico-spinal motor neuron (CSMN)-specific genes, Arlotta et al. confirmed that the transcription factor Ctip2 is expressed in all subcerebrally-projecting neurons and is required for CSMN axon extension to the spinal cord and postnatal maintenance of these axons (Arlotta et al., 2005). Satb2, another transcription factor, is an important marker of corticocortical connectivity. Satb2 mutant brains have a thinner cortex (~15% in CP and ~20% in intermediate zone) than found in wild-type brains, as a consequence Satb2 null mice die at birth (Alcamo et al., 2008; Britanova et al., 2008). Reduced cortical thickness is due to defects in migration, proven with BrdU-labeling. Satb2 null mouse axons do not cross to the other hemisphere via the corpus callosum, but rather join CSMN in projecting subcerebrally. In mutant brains, ectopic Ctip2 expression was seen in upper layer neurons based on BrdU-tracing at E15.5; WT neurons cycling at this time do not express Ctip2, but Satb2-/- neurons cycling at this time do express Ctip2 (Alcamo et al., 2008). This resulted in ~27% more cells projecting subcerebrally to the cerebral peduncle (a fascicle containing axons projecting corticopontine, corticobulbar, and corticospinal) (Britanova et al., 2008). Satb2 is likely a transcriptional repressor of Ctip2 via histone acetylation (Alcamo et al., 2008), by assembling the NURD chromatin complex (Britanova et al., 2008).

In order to better understand callosal projections, further study of cortical transcription factor Lmo4 would be informative as, unlike Satb2, it is expressed in all callosal neurons (Arlotta et al., 2005). In the Satb2 knockout, Lmo4 expression is reduced in layers V and VI mediodorsally and elevated in the intermediate zone (expression patterns were analyzed at E18.5 due to Satb2 -/- lethality) (Alcamo et al., 2008). This could imply that Lmo4 helps to define a subpopulation of corticocortical neurons in a majority corticofugal environment.

Post-transcriptional regulation was described in a previous section of this review as partly responsible for the genesis of a heterogeneous progenitor pool. This further extends into the maturation phase. A recent screen of post-natal cells in the mouse visual cortex found that 320 genes, and specifically 567 exons, were subject to differential mRNA processing (i.e., polyadenylation, alternative splicing) between diverse subpopulations (Tasic et al., 2016). Within the somatosensory cortex, ~16% (1,646) of genes were subject to differential patterns of alternative splicing (Belgard et al., 2011). These results suggest that post-transcriptional processing remains abundant throughout and beyond the progenitor period- possibly as a mechanism for fate maintenance.

*Lmo4* mRNA is a target of a lower-layer specific RBP, HuD (Chen et al., 2007; DeBoer et al., 2014). HuD, one of the Hu antigens, is a neuronal lineage-specific RBP (Okano and Darnell, 1997). In addition to being expressed in a subpopulation of lower layer neurons in the adult murine neocortex (DeBoer et al., 2014), it has been found in human IPs as well (Pollen et al., 2015). Constitutive knockout of this protein results in a specific loss of Tle4+ lower layer populations (~12% reduction compared to WT), but does not significantly affect upper layer populations (DeBoer et al., 2014). HuD is known to be important for post-mitotic maturation of neurons, and specifically for formation of dendritic trees. miR-375 has been found to inhibit dendritic differentiation by targeting *HuD* mRNA (Abdelmohsen et al., 2010). Accordingly, *in vivo*, HuD depletion resulted in decreased dendritic complexity (DeBoer et al., 2014). Considering that HuD is widely expressed in lower layers of the cortex, including neurons which project axons along the corticospinal tract, it is not surprising that HuD loss affects motor performance on the rotorod (Akamatsu et al., 2005).

One of the most striking abnormalities in HuD mutants is their propensity for sound-induced seizures: when confronted with a metallic stimulus (i.e., rapid jingling of a metal object), ~63% of mutants convulsed and of these, ~38% of these events resulted in death. A battery of behavioral testing also revealed that HuD mutants spent more time in the open arms of the elevated plus maze. Mutants also spent more time engaging in low-energy activities such as standing still and less time engaging in high-energy activities such as running (DeBoer et al., 2014). Overall, the phenotypes seen in HuD mutants demonstrate that interfering in an RBP will have salient disruption of specific aspects of neuronal development such as dendritic maturation and circuit integration.

HuR is a ubiquitously-expressed RBP and another of the ELAVL proteins. It has been found to be expressed in NEC, RG, IPs, and neurons (Garcia-Dominguez et al., 2011; Kraushar et al., 2014). In order to study the role of HuR in neocortical development, HuR conditional knockout (*Emx1-Cre* driven knockout) neocortex was dissected at E13 and postnatal day 0 and compared to wildtype cortices in a recent study (Kraushar et al., 2014). The neocortex was processed through polysome-fractionation, mRNA isolation from the fractions, and finally RNAseq of fraction-specific mRNA. Kraushar et al. found that transcripts expressed in layers II/III and V were disproportionately impacted by HuR deletion at both ages and both in mono- and polysomes. Strikingly, by age P0, layer II/III transcripts were enriched in HuR conditional knockout cortices while layer V transcripts were decreased in the HuR conditional knockout cortices (Kraushar et al., 2014). Transcriptomic profiling has identified significant gene expression overlap between layers II/III and V (Hoerder-Suabedissen et al., 2013). In this context, results obtained with HuR conditional knockout are tantalizing because of HuR's widespread expression across all the neocortical layers, suggesting differential actions between upper and lower layers. An extended comparison across layers in this mutant for AS variants could be revealing.

In order to investigate a specific example of the upper/lower layer bias of HuR, two of its regulated transcripts were examined: *Foxp1* and *Foxp2*. Both are members of the Forkhead box family of transcription factors; these are distinct from RBFOX-1/2, RBFOX-3 is also known as NeuN (Figure 1), which are the mammalian homologs of the *C. elegans* RBP Fox-1. Mammalian FOXP2 is a transcription factor expressed in a subpopulation of cells in layer VI (Ferland et al., 2003) that project corticothalmaically (Sorensen et al., 2015). It is expressed in post-mitotic cells and has been implicated in Autism Spectrum Disorders (ASDs) (Vernes et al., 2011). It has been shown that *Foxp2* mRNA translation requires the RBP HuR during prenatal cortical development (Popovitchenko et al., 2016). *Foxp2* was identified alongside *Foxp1* as bound targets of HuR in a RIP-ChIP (RNA immunoprecipitation coupled with a microarray) screen. In the HuR conditional knockout, FOXP2 protein was absent when it normally should have been present in the neonatal brain (P0), however, *Foxp2* mRNA levels in HuR conditional knockout were comparable to levels in wildtype, demonstrating the reliance of the *Foxp2* transcript on post-transcriptional processes to enact its translation. Interestingly, the related protein FOXP1 was precociously expressed in the appropriate subpopulation of cells (Layers III-V), suggesting that HuR is important for translational-repression of this transcription factor (Figure 5, step 6). Differential phosphorylation was proposed as a likely mechanism for HuR's differential treatment of these two transcripts, and demonstrated with an *in vitro* translational assay (Popovitchenko et al., 2016), though no specific kinase was identified. It would be informative to further investigate HuR's control of neuronal maturation and layer formation; specifically as to whether dysregulation of Foxp2 translation confers an anatomical, i.e., cortico-thalamic connectivity, or behavioral, i.e., murine ultra-sonic vocalizations, phenotype.

## Conclusions

Proper lamination requires that progenitor cells give rise to a neuron at a certain time point during development, that the immature neuron can migrate away from its birth place and past other cells on its way, and finally that the immature neuron can stop to take its proper place and adopt a mature identity as characterized by dendritic and axonal patterns, electrophysiology, and gene expression. It is becoming clear that both transcriptional and post-transcriptional mechanisms guide the progression of each of these steps, crucial to neocortical laminar identity.

Fate is often exquisitely linked to functional gene expression. For example, we can confidently identify a RG because it expresses Pax6. However, relying on one molecule for identification leaves the possibility open that co-expressing subpopulations (i.e., Pax6 and Tbr2) will be improperly identified at the progenitor (Telley et al., 2016) and the post-mitotic levels (Handel et al., 2016). Furthermore, a recent single-cell RNA seq screen of iPSCs found that *Bcl11b/Ctip2* was co-expressed with *Brn2* in a subpopulation of cells; and that this is recapitulated in neurons from human fetal and adult brains (Handel et al., 2016). Therefore, the use of several overlapping factors will lead to better understanding of the progenitor and post-mitotic subpopulation heterogeneity.

Characterization of transcription factors in specific subpopulations of neurons was analyzed at depth and several important determinants of cell fate have been identified. In particular, the onset of studies using RNAseq, which allows for unbiased sequencing of transcripts and subsequently the discovery of processes occurring post-transcriptionally, such as alternative splicing or unanticipated identification of novel non-coding transcripts, is beginning to reveal the importance of mRNA processing across cell types. Alternative splicing and translational repression, such as that enacted by lncRNAs, are emerging as two distinct mechanisms by which regulation occurs in addition to the localization of transcripts by RBPs and translational suppression/repression through binding. Also of note is the heavy involvement and dependence of posttranscriptional regulation with cell-cycle control (Abdelmohsen et al., 2008; Filippova et al., 2012; Boulay et al., 2014; Duggimpudi et al., 2015), regulation of which is repeatedly found to correspond to state-transitions.

Unlike TFs, post-transcriptional regulators often have ubiquitous expression patterns and are not bound by the neat compartments of gene expression that characterize the layers (Figure 2C). Thus, how can they fit into our understanding of laminar development? Differential phosphorylation states that are controlled by distinct kinases may play a role in translational specificity of bound mRNAs (Popovitchenko et al., 2016). In addition, competitive roles of distinct RBPs, miRNAs and their targets may further contribute to the diversity (Gardiner et al., 2015). Interestingly, genes that did not have laminar specificity were more likely to be important in development in an RNA-seq screen of post-natal brains (Belgard et al., 2011). Though largely speculative at this point, this observation could be a clue to the over-arching importance of RNA processing during development.

Further exploration into post-transcriptional determinants of cell fate is demonstrably needed. With every step of mRNA processing open to spatiotemporally specific post-transcriptional intervention, the full contribution of post-transcriptional processes to laminar characterization remains to be discovered.

## Author contributions

TP and MRR conducted literature searches, designed figures, and completed writing of the manuscript.

### Conflict of interest statement

The authors declare that the research was conducted in the absence of any commercial or financial relationships that could be construed as a potential conflict of interest.
